# Effects of Pentoxifylline on Peri-Implant Capsule Cellularity and Thickness: A Controlled Experimental Study in a Rat Silicone Implant Model

**DOI:** 10.3390/ijms27156779

**Published:** 2026-07-29

**Authors:** Tevfik Satir, Armin Kraus, Ibrahim Güler

**Affiliations:** 1 Center for Dermatosurgery, St. Josefskrankenhaus Heidelberg, Academic Teaching Hospital of the Medical Faculty Mannheim, Heidelberg University, 69115 Heidelberg, Germany; t.satir@st.josefskrankenhaus.de; 2 Department of Plastic and Reconstructive Surgery, Marmara University Hospital, 34899 Istanbul, Turkey; 3 Department of Plastic, Aesthetic and Hand Surgery, Otto-von-Guericke University, 39120 Magdeburg, Germany; armin.kraus@med.ovgu.de; 4 Department of Health Management, Friedrich-Alexander-Universität Erlangen-Nürnberg, 90403 Nürnberg, Germany

**Keywords:** pentoxifylline, capsular contracture, peri-implant capsule, fibrosis, TGF-β1, myofibroblast, profibrotic signaling, antifibrotic therapy, tissue remodeling, rat model

## Abstract

Capsular contracture is a fibrotic foreign-body response to silicone implants, driven primarily by TGF-β1-mediated myofibroblast activation and profibrotic signaling, with no established pharmacological prophylaxis. Pentoxifylline (PTX), an antifibrotic agent acting on these pathways, has rarely been evaluated for peri-implant capsule formation. Twenty-eight male Sprague Dawley rats received a 2 cm × 1 cm smooth silicone block in a dorsal sub-panniculus carnosus pocket. After pre-specified exclusions, 20 animals were analyzed in two equal groups: control (saline) and PTX (25 mg/kg/day intraperitoneally) for six weeks. Intracapsular fibroblast/myofibroblast density and capsule thickness were quantified by H&E histology with digital image analysis. PTX reduced fibroblast/myofibroblast density by 35.7% (36.28 ± 9.80 vs. 56.46 ± 16.69 cells/HPF; *p* = 0.004; d = 1.47). Capsule thickness was reduced by 33.0% (340.10 ± 199.88 vs. 507.78 ± 170.72 µm; d = 0.90), which did not reach significance (Student’s t *p* = 0.059); the Mann–Whitney U sensitivity analysis gave *p* = 0.045. Systemic PTX reduced intracapsular fibroblast/myofibroblast density, with a directionally consistent but statistically inconclusive secondary signal for capsule thickness. Consistent with the antifibrotic profile of PTX, these findings identify a candidate signal warranting confirmation in a mechanistically powered follow-up, within the limitations of a single-dose, single-sex, H&E-based design.

## 1. Introduction

Silicone implants are a cornerstone of modern reconstructive and aesthetic surgery, with breast augmentation alone accounting for several hundred thousand procedures performed annually. The biological response to a permanent silicone foreign body is the formation of a periprosthetic fibrous capsule, an organized scar produced by the late phase of wound healing. When this capsule undergoes pathological remodeling and contraction it results in capsular contracture—the most frequent long-term complication of implant-based breast surgery—with reported incidence ranging from approximately 2.8% to 20% in primary augmentation and substantially higher rates after radiotherapy [1,2,3,4].

Despite extensive investigation over many decades, the molecular drivers of pathological capsule formation remain incompletely defined. Current evidence implicates a multifactorial process involving subclinical bacterial biofilm, mechanical and chemical stimulation by the implant surface, persistent foreign-body type inflammation, and dysregulated myofibroblast activity driven primarily by transforming growth factor beta 1 (TGF-β1) and downstream Smad and non-Smad signaling. Mechanosensing through Piezo1 has more recently been implicated as an upstream amplifier of this fibrotic program. Within the mature capsule, fibroblasts produce the dominant collagenous matrix, while alpha smooth muscle actin (α-SMA)-positive myofibroblasts mediate active contraction; the density of both cell populations correlates with clinical capsule firmness [2,3,5,6,7].

At the molecular level, peri-implant capsule formation unfolds as a temporally ordered cascade that begins with a foreign-body inflammatory phase and matures into a fibrotic one. After implantation the device surface is coated by adsorbed plasma proteins, triggering the recruitment of neutrophils and macrophages that, on a permanent and non-degradable surface, undergo frustrated phagocytosis, fuse into foreign-body giant cells and sustain a chronic release of pro-inflammatory and pro-fibrotic mediators, including tumor necrosis factor-α (TNF-α), interleukin-1β (IL-1β), interleukin-6 (IL-6) and interleukin-8 (IL-8) [2,4,8]. Against this inflammatory background, TGF-β1 acts as a central master switch: signaling predominantly through phosphorylation of Smad2/3, it drives the activation of resident fibroblasts and their transition into contractile myofibroblasts, and experimental interruption of this axis—for example, through loss of Smad3 function—has been shown to yield thinner and more regularly organized capsules [3]. The resulting α-SMA-positive myofibroblasts generate the contractile force that remodels the capsule while depositing a dense type I and type III collagen matrix; the balance between matrix metalloproteinases (MMPs) and their tissue inhibitors (TIMPs) governs this remodeling, with dysregulation of this balance contributing to matrix accumulation, as described in other progressive fibrotic disorders [3]. The intensity of this response is not uniform, and the local density of fibroblasts and myofibroblasts at the implant–tissue interface correlates with the clinical severity of contracture, linking a cellular readout to the graded clinical phenotype [3]. Superimposed on this core axis, an adaptive immune component characterized by a T helper 1 and T helper 17 skew with reduced regulatory T-cell suppression in higher-grade contracture, and mechanotransduction through the stretch-activated ion channel Piezo1, which converts implant-related increases in tissue stiffness into intracellular calcium signaling, further amplify the fibrotic program [6,7]. Across these converging inputs the TGF-β1-driven fibroblast-to-myofibroblast axis represents the common final pathway of pathological capsule formation, providing the rationale for pharmacological strategies that target profibrotic signaling at the cellular level [2,5,8]. Notably, capsular myofibroblasts have been reported to express estrogen receptors, with their contractile activity increasing in response to circulating 17β-estradiol, one of several reasons the fibrotic response is preferentially studied under controlled hormonal conditions [2,3].

A wide range of preventive strategies have been investigated, including textured implant surfaces, pocket irrigation with antiseptics or antibiotics, intraluminal or intracapsular steroids, leukotriene receptor antagonists (LTRAs), vitamin E and vitamin A, mitomycin C, hyaluronic acid, glucosamine and chondroitin, transforming growth factor beta inhibitor peptides, acellular dermal matrix (ADM) wrapping, fat grafting, and adipose-derived stem cells (ADSCs). Recent systematic reviews and meta-analyses summarize this heterogeneous literature and underline that no pharmacological intervention has yet achieved a durable, clinically validated reduction in capsular contracture as a stand-alone strategy. A 2025 systematic review of murine pharmacological interventions on miniaturized breast implants identified 29 studies of pharmacological agents and surface treatments without reaching a consensus on a preferred agent [2,5,8,9,10,11,12,13].

Against this background, drug repurposing offers an attractive translational route: an established agent with a known safety profile and existing manufacturing infrastructure can move from preclinical signal to clinical evaluation considerably faster than a new chemical entity. Pentoxifylline (PTX) is a methylxanthine derivative and non-selective phosphodiesterase (PDE) inhibitor in continuous clinical use since the 1970s for peripheral and cerebrovascular disease, characterized by low cost, broad availability and an established safety profile. Beyond its hemorheological action, PTX possesses well-documented antifibrotic activity, with in vitro inhibition of fibroblast proliferation and collagen synthesis demonstrated in keloid, scleroderma, morphea and burn-scar fibroblasts as well as in vascular smooth muscle cells under platelet-derived growth factor (PDGF) and TGF-β stimulation, and with more recent mechanistic links to suppression of nuclear factor kappa B (NF-κB) signaling, downregulation of serpin family E member 1 (SERPINE1), inhibition of TGF-β1-induced epithelial–mesenchymal transition, and engagement of an Akt/FoxO/p27Kip1 axis with downregulation of Collagen type I, Collagen type III and α-SMA in hypertrophic scar fibroblasts [14,15,16,17,18]. Clinically, the combination of PTX with α-tocopherol (vitamin E) has produced regression of established radiation-induced fibrosis in a randomized placebo-controlled trial and in subsequent observational and phase II work, with mixed results in larger randomized prevention trials, and a single-arm prophylactic study in post-mastectomy implant reconstruction with adjuvant radiotherapy reported low revision rates for contracture and no implant losses with the same regimen [19,20,21,22,23].

Despite this antifibrotic profile—and despite the central role of fibroblast proliferation in capsule biology—dedicated in vivo characterization of systemic PTX in a controlled silicone implant model remains scarce, and the agent is not among those cataloged in a systematic murine review [9]. The present study addresses this gap. We hypothesized that daily systemic administration of PTX at 25 mg/kg over six weeks, the interval after which a mature periprosthetic capsule is reliably observed in the rat [24], would reduce both capsule thickness and intracapsular fibroblast/myofibroblast density compared with saline-treated controls.

## 2. Results

### 2.1. Animal Survival and Macroscopic Findings

Of the 28 enrolled animals allocated 14 per group, eight were excluded from histopathological analysis as pre-specified, in an identical distribution across the two groups: four died in the perioperative period, before the first treatment dose (two per group), and four developed macroscopically evident peri-implant hematomas with capsule thickening at explantation, leaving 10 animals per group for analysis. No animals developed wound infection, dehiscence or other complications during the six-week observation period. Body weight, food and water intake remained stable in all surviving animals.

**Figure 1:**
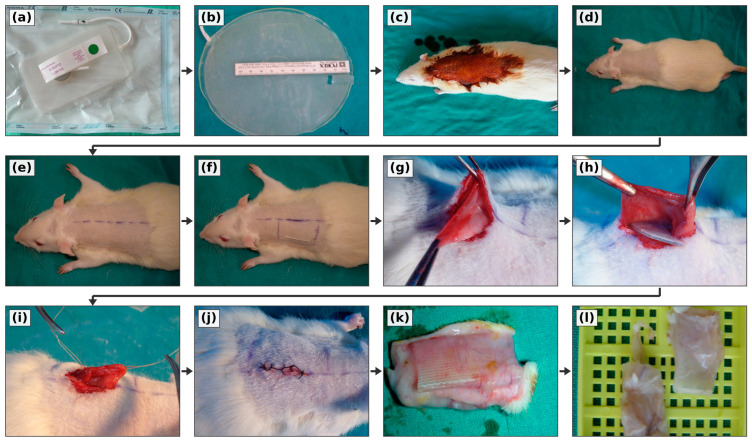
Macroscopic surgical workflow of the dorsal sub-panniculus carnosus pocket model in the rat. Panels are representative images of the procedure. (**a**) Sterile silicone tissue expander used as the source material. (**b**) Silicone sheet harvested from the expander, from which 2 cm × 1 cm blocks were prepared for implantation. (**c**) Antiseptic preparation of the dorsal field. (**d**) Anesthetized male Sprague Dawley rat in prone position with the prepared dorsal field. (**e**) Shaved operative field with marking of the planned vertical thoracodorsal incision. (**f**) Marking of the implant footprint adjacent to the incision line. (**g**) Blunt dissection of the sub-panniculus carnosus pocket through the vertical incision. (**h**) Insertion of the 2 cm × 1 cm silicone block into the subcutaneous pocket. (**i**) Approximation of the deep layer with absorbable sutures. (**j**) Skin closure with interrupted sutures. (**k**) Implant retrieved at six weeks showing the surrounding peri-implant fibrous capsule on the silicone surface. (**l**) Excised capsule samples placed in a tissue cassette for formalin fixation and paraffin embedding prior to histological analysis.

On macroscopic examination at week 6 (Figure 1), control group capsules appeared as a fascia-like white-yellow shell adherent to the surrounding tissue, with hyperemic vascularized areas. Capsules in the PTX group were thinner, smoother, freely mobile against the surrounding tissue and macroscopically less vascularized.

### 2.2. Fibroblast/Myofibroblast Density

**Figure 2:**
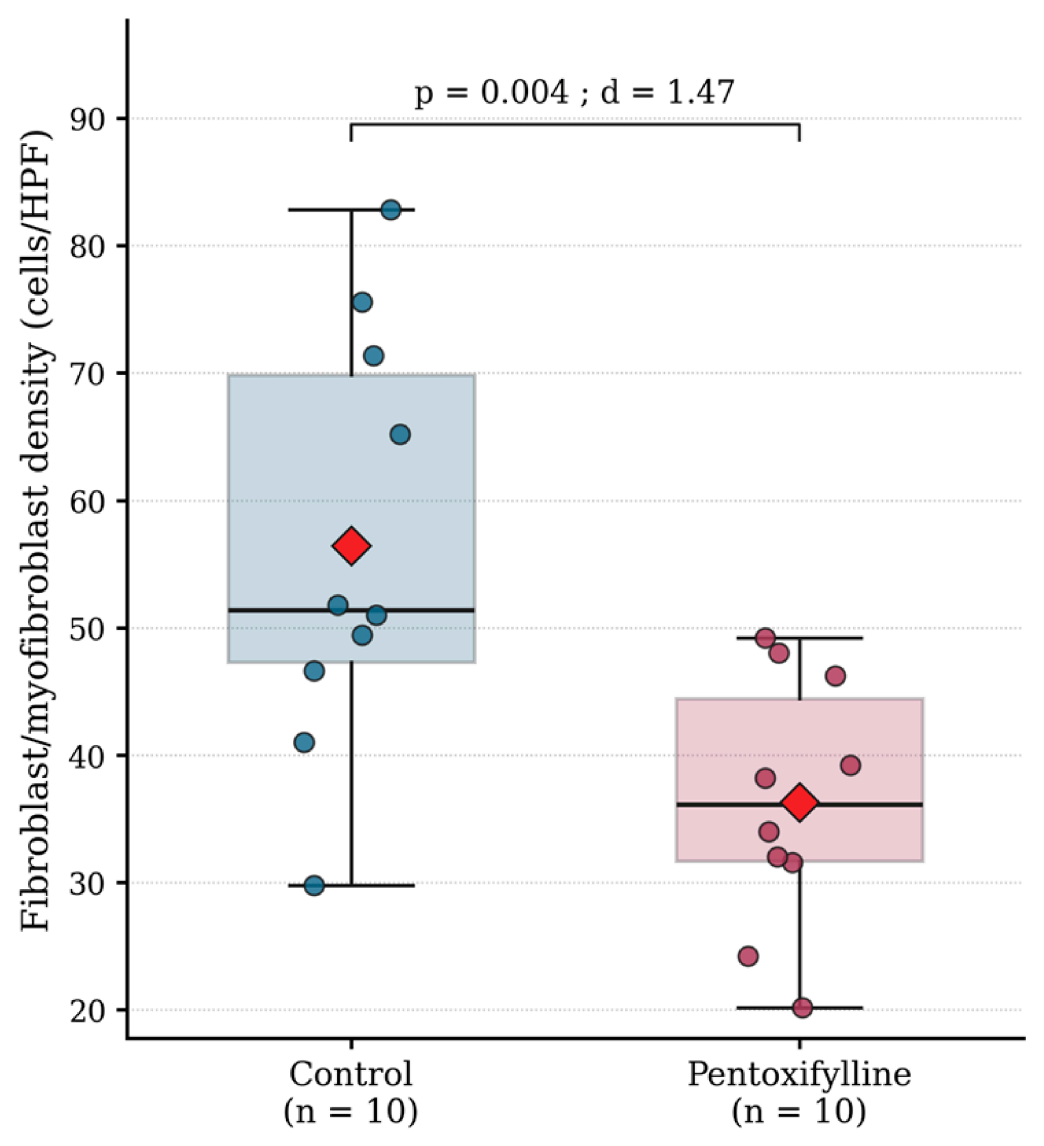
Distribution of intracapsular fibroblast/myofibroblast density in the control and PTX groups (*n* = 10 per group). Boxes indicate interquartile range with the median as a horizontal line; whiskers extend to the most extreme values; group means are shown as red diamond markers. Individual animal values are overlaid as jittered circles. The bracket indicates the parametric Student’s *t*-test *p*-value and Cohen’s d effect size.

**Figure 3:**
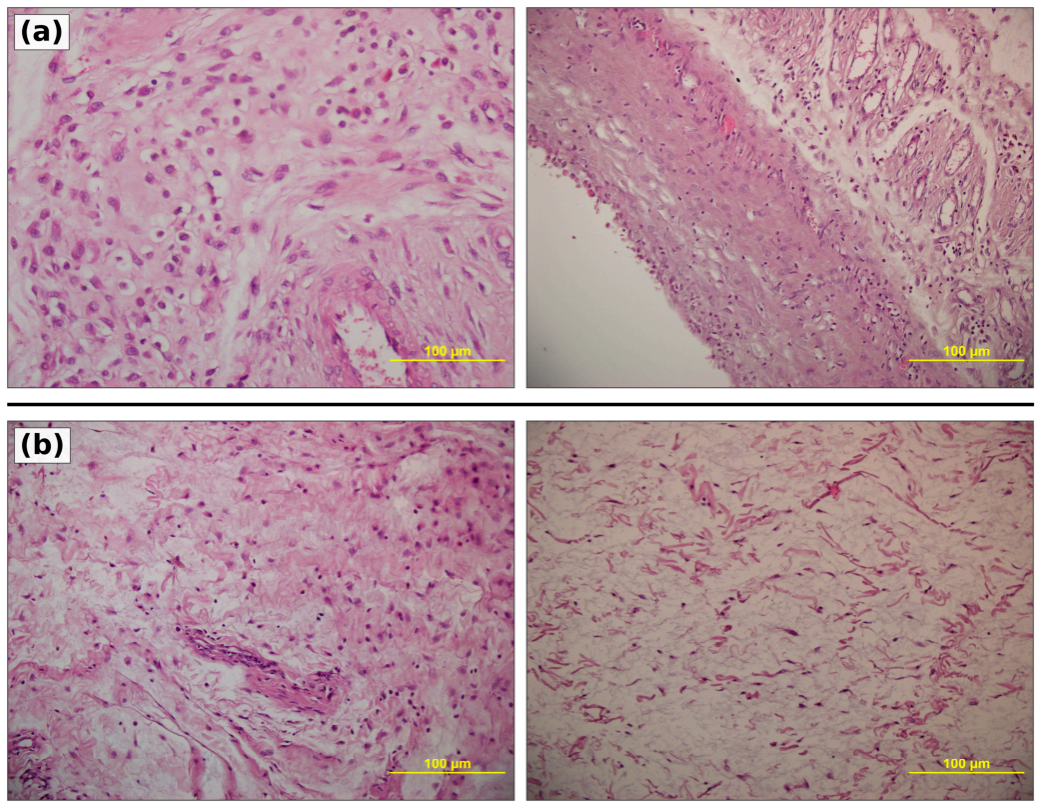
Representative photomicrographs of the peri-implant capsule structure used for fibroblast/myofibroblast counting (hematoxylin and eosin, original magnification ×400; objective ×40; scale bar 100 µm). (**a**) Control group. (**b**) PTX group: notably reduced lymphocyte infiltration and reduced capillary proliferation compared with the control group.

Mean fibroblast/myofibroblast density was 36.28 ± 9.80 cells per high-power field (HPF) in the PTX group (range 20.20–49.20) versus 56.46 ± 16.69 cells/HPF in the control group (range 29.80–82.80). The mean difference of 20.18 cells per high-power field (95% confidence interval [CI] 7.10–33.26) corresponds to a 35.7% reduction in the treated group, with a large effect size (Cohen’s d = 1.47). The difference was significant by Student’s *t*-test (*p* = 0.0040), and the Mann–Whitney U sensitivity analysis was concordant (*p* = 0.0058). The group distribution is shown in Figure 2, and representative high-power photomicrographs in Figure 3.

### 2.3. Capsule Thickness

**Figure 4:**
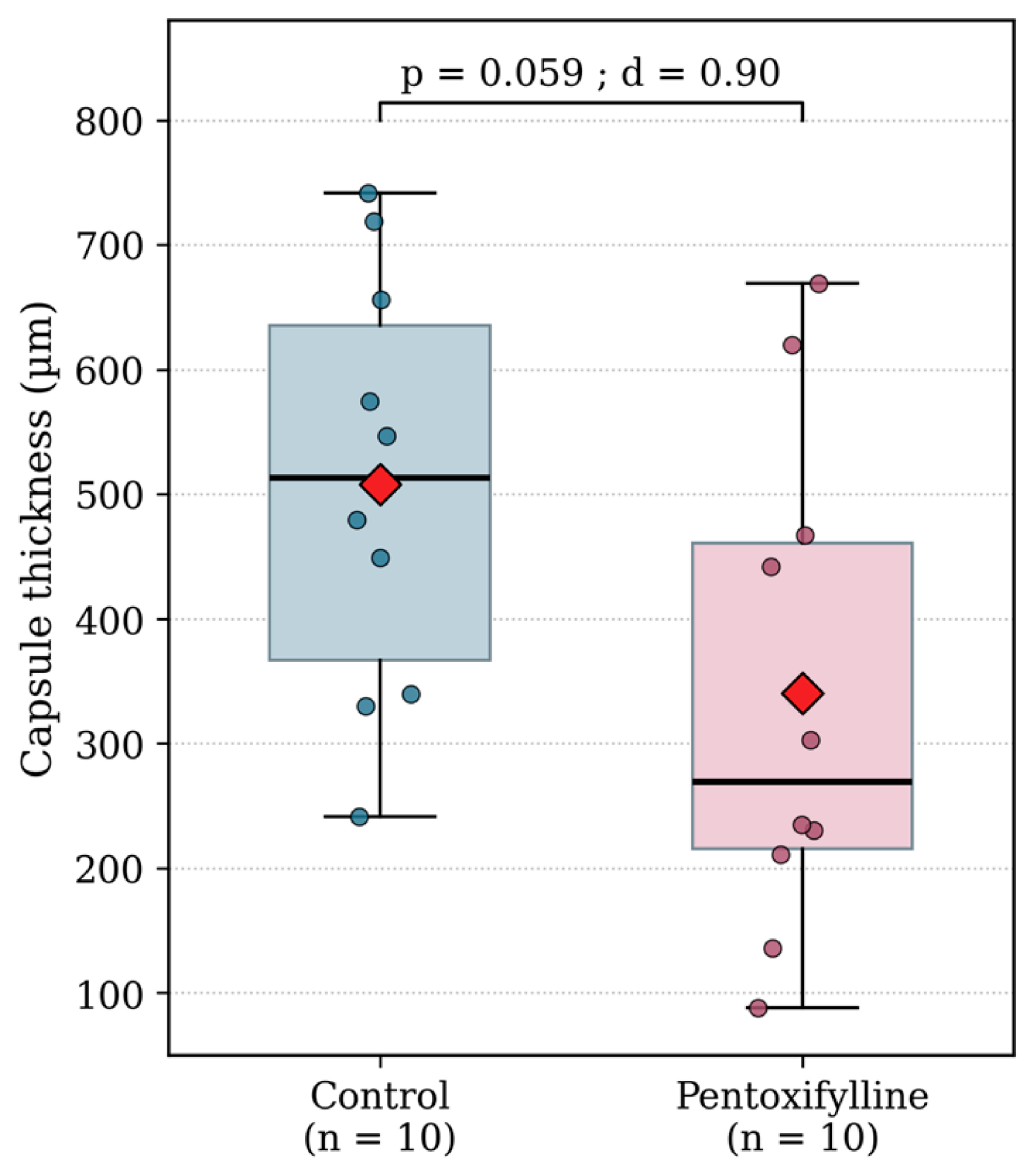
Distribution of peri-implant capsule thickness in the control and PTX groups (*n* = 10 per group). Boxes indicate interquartile range with the median as a horizontal line; whiskers extend to the most extreme values; group means are shown as red diamond markers. Individual animal values are overlaid as jittered circles. The bracket indicates the Student’s *t*-test *p*-value and Cohen’s d effect size.

**Figure 5:**
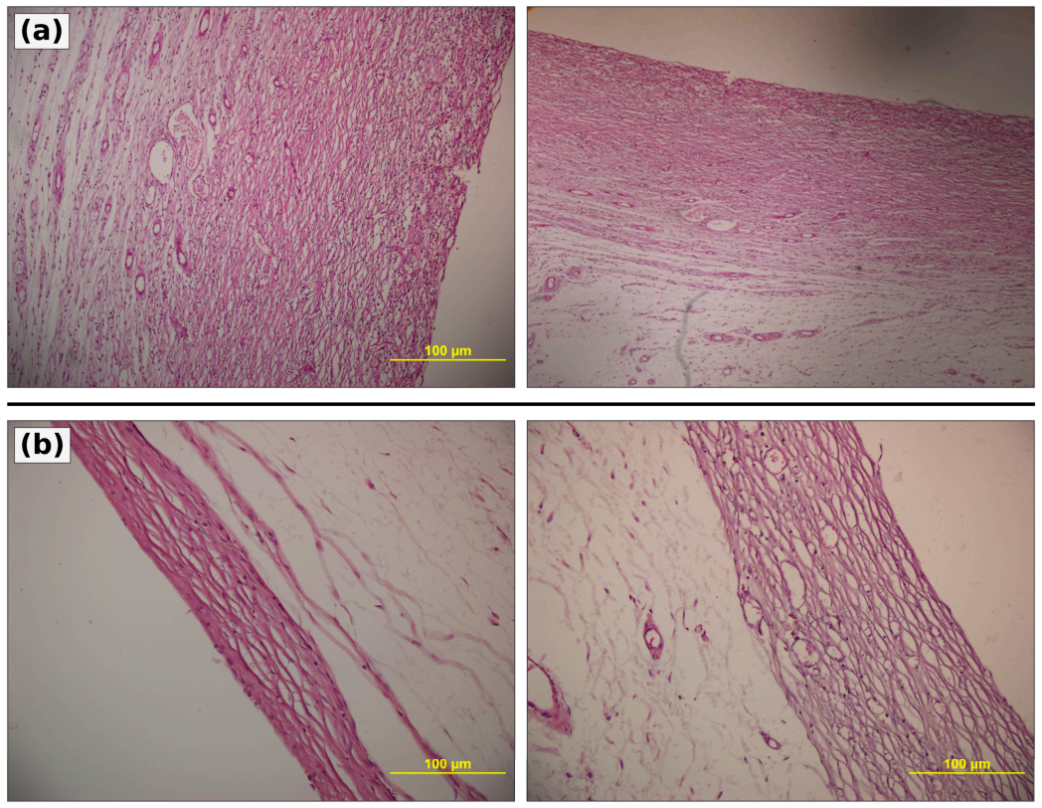
Representative photomicrographs of the peri-implant capsule used for thickness measurement (hematoxylin and eosin, original magnification ×100; objective ×10; scale bar 100 µm). (**a**) Control group: a thick, cellular, densely collagenous capsule. (**b**) PTX group: a thinner, less cellular capsule with looser fiber architecture compared with the control group.

Mean capsule thickness was 340.10 ± 199.88 µm in the PTX group (range 87.70–669.16 µm) versus 507.78 ± 170.72 µm in the control group (range 241.19–741.47 µm). The mean difference of 167.69 µm corresponds to a 33.0% reduction in the treated group, with a large effect size (Cohen’s d = 0.90). The 95% CI for the mean difference (−7.26 to 342.63 µm) included zero, and the parametric Student’s *t*-test did not reach the conventional significance threshold (*p* = 0.0588). The Mann–Whitney U sensitivity analysis yielded *p* = 0.0452. The group distribution is shown in Figure 4, and representative low-power photomicrographs in Figure 5. Summary statistics for both endpoints are presented in Table 1.

### 2.4. Other Histopathological Features

Although not formally quantified, sections from the PTX group showed notably reduced lymphocytic infiltration and reduced capillary proliferation compared with control sections. These descriptive observations, without quantitative validation, are consistent with the documented anti-inflammatory and antiangiogenic activity of PTX but should be interpreted with caution given the absence of formal scoring or immunohistochemical confirmation.

## 3. Discussion

In this controlled rat model, six weeks of daily systemic PTX at 25 mg/kg produced a robust 35.7% reduction in intracapsular fibroblast/myofibroblast density (Cohen’s d = 1.47), with a 95% CI that excluded the null effect and significance by Student’s *t*-test (*p* = 0.0040), with the Mann–Whitney U sensitivity analysis concordant (*p* = 0.0058). A parallel 33.0% reduction in mean capsule thickness was observed (Cohen’s d = 0.90), which did not reach significance by Student’s *t*-test (*p* = 0.0588) and had a CI that crossed zero, although the Mann–Whitney U sensitivity analysis reached the threshold (*p* = 0.0452). Macroscopically, capsules in the treated group were thinner, less adherent and less vascularized. Together these findings are consistent with a primarily cellular pattern of effect of PTX at the peri-implant interface, with downstream tissue-level effects on capsule thickness that are directionally consistent in magnitude but more variable across animals. The magnitude of the cellularity reduction is large by conventional benchmarks (d > 0.8). PTX is not among the agents cataloged in a systematic review of pharmacological interventions in murine implant models, and appears to have received little dedicated attention as a systemic intervention against the silicone-implant capsule [9].

These findings sit within the broader literature on pharmacological prevention of capsular contracture that has produced no agent of established and durable clinical efficacy as monotherapy. Among clinical interventions, LTRAs (montelukast, zafirlukast) carry the most consistent supportive signal, although recent meta-analyses report heterogeneous subgroup effects and limited randomized data [12,25,26,27,28]. ADM wrapping reduces contracture rates in pooled clinical analyses and is the most established device-based adjunct, although matrix type strongly influences efficacy [10,29,30]. Adipose-derived stem cell (ADSC) therapy and cell-assisted hybrid breast reconstruction, fat grafting and various antifibrotic agents have shown promise in experimental and early clinical work without yet maturing into accepted clinical practice [5,9,11,13,31,32,33,34,35]. In contrast to device-based or technique-based strategies, PTX represents a systemic pharmacological approach characterized by low cost, broad availability and an established multi-decade safety profile, and offers documented translational continuity with the prophylactic clinical study by Cook et al. in implant-based breast reconstruction under adjuvant radiation therapy [23].

**Figure 6:**
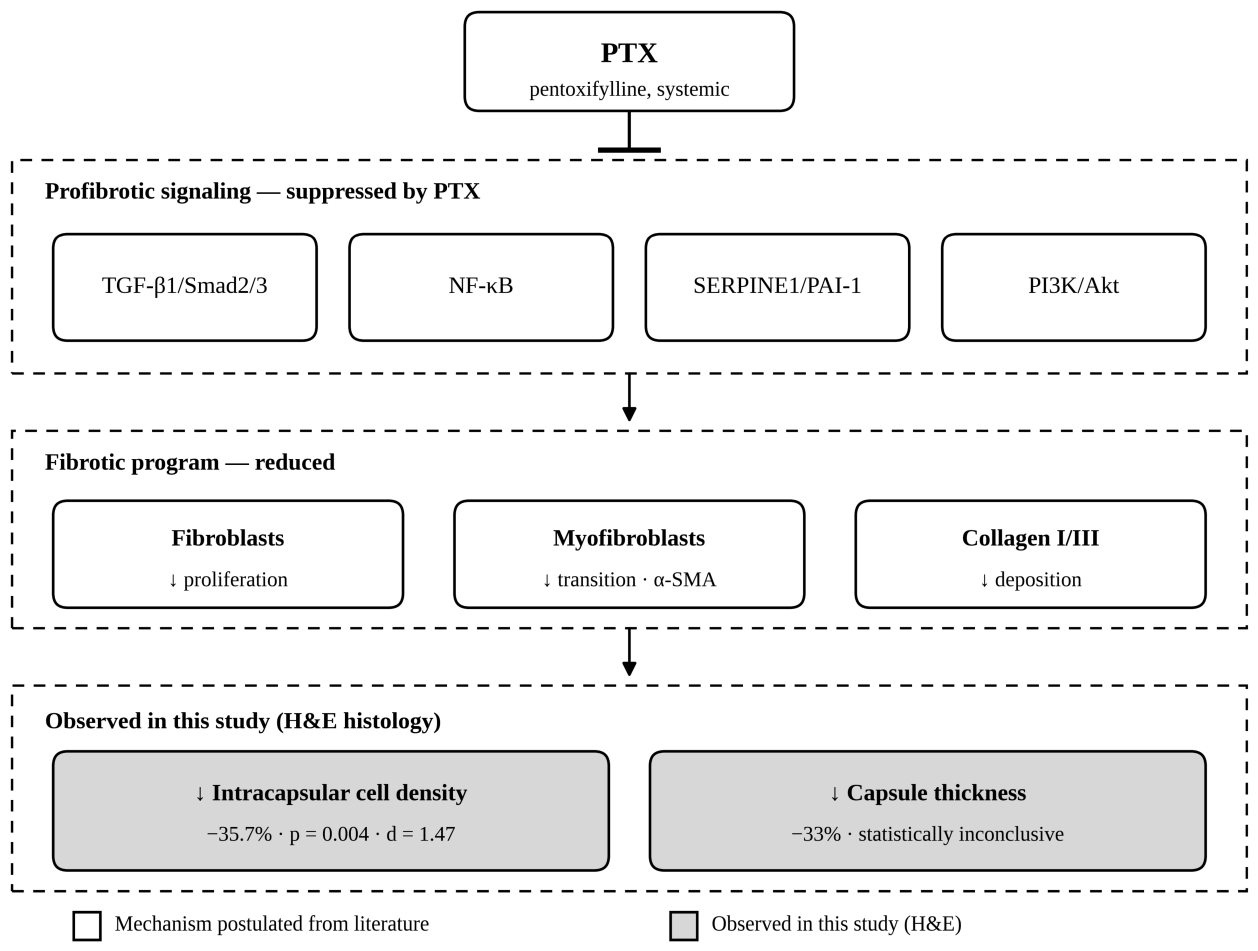
Proposed molecular mechanism of the antifibrotic effect of PTX on peri-implant capsule formation. Schematic summary of the signaling pathways through which PTX is reported to act, postulated on the basis of the established literature [5,6,7,8,14,15,16,17,18,36,37,38].

The biological plausibility of an antifibrotic effect of PTX on the periprosthetic capsule, and the cellular pattern observed in the present data, are coherent with the increasingly mechanistic recent literature (Figure 6). PTX inhibits fibroblast proliferation and collagen synthesis in vitro across multiple fibrotic models including keloid, scleroderma, morphea and burn-scar fibroblasts, with mechanistic links to suppression of NF-κB signaling, downregulation of SERPINE1 and inhibition of TGF-β1-induced epithelial–mesenchymal transition, as well as engagement of an Akt/FoxO/p27Kip1 axis with downregulation of Collagen type I, Collagen type III and α-SMA in hypertrophic scar fibroblasts [14,15,16,17,18,36,37]. Because TGF-β1 and α-SMA-positive myofibroblasts are central to peri-implant capsule formation, and because intracapsular myofibroblasts derive from resident fibroblasts and are the principal mediators of capsule contraction, a reduction in fibroblastic activity is biologically expected to indirectly reduce the myofibroblast pool that drives contractile remodeling [5,6,7,8,38]. This logic offers a coherent mechanistic explanation for the pattern observed in our data: a primary cellular effect on the proliferation and accumulation of capsular fibroblasts, with thickness reduction following as a downstream tissue-level consequence. The clinical study by Cook and colleagues, in which prophylactic PTX plus vitamin E was used after implant reconstruction with adjuvant chest-wall radiotherapy and showed low revision rates with no implant losses, provides a supportive translational signal in the most challenging clinical context, namely irradiated breast reconstruction [23].

The histological endpoints quantified in the present study can be situated directly within this molecular framework. The intracapsular fibroblast/myofibroblast population counted on hematoxylin and eosin (H&E) represents the cellular endpoint of TGF-β1- and Smad-driven fibroblast activation and myofibroblast differentiation, the α-SMA-positive myofibroblast being the effector cell of this cascade, while capsule thickness reflects the type I and type III collagen matrix that the same signaling deposits [3,5,8]. Because the number of capsular fibroblasts and myofibroblasts correlates with the clinical grade of contracture, a reduction in this cell population is a biologically meaningful endpoint rather than an incidental morphometric observation [3]. The reduction in intracapsular cell density observed under PTX can therefore be read as a tissue-level correlate of precisely the profibrotic program, encompassing NF-κB signaling, SERPINE1 expression, TGF-β1-induced cellular transition and the fibroblast-to-myofibroblast switch, that PTX is reported to suppress in vitro [14,15,16,17,18,36,37]. We emphasize, consistent with the limitations set out below, that no immunohistochemical or molecular assays were performed in this work; the H&E-based cell count is a morphology-based tissue correlate of this signaling rather than a direct measurement of any individual molecular mediator, and the composite term fibroblast/myofibroblast is retained throughout for this reason. Interpreted within this constraint, the data show an internally coherent pattern: the robust and statistically concordant effect on cellularity is consistent with an action of PTX on the proliferating fibroblast pool that lies upstream in the cascade, whereas the directionally consistent but statistically inconclusive thickness signal is compatible with collagen-matrix accumulation representing a more distal and multifactorial endpoint that is inherently more variable and, at the present group size, underpowered. This reading does not establish a molecular mechanism in vivo, which would require the dedicated immunohistochemical and collagen-organization readouts outlined below, but it aligns the observed cellular signal with the established molecular pharmacology of the drug. Consistent with this, whole-genome transcriptomic analysis of contracted human capsules, validated by immunohistochemistry, has identified a characteristic molecular signature of fibrosis, with upregulation of IL-8 and MMP-12 and downregulation of the tissue inhibitor of metalloproteinases TIMP4, indicating that the cellular phenotype scored histologically reflects a defined underlying transcriptional program [8].

More broadly, peri-implant capsule formation is increasingly understood as a signaling network rather than a single linear axis, and the antifibrotic targets attributed to PTX intersect several of its nodes. The chronic foreign-body response is sustained by macrophages whose balance between a pro-inflammatory M1 and a pro-fibrotic M2 phenotype shapes the fibrotic outcome, together with a cytokine milieu of TNF-α, IL-1β, IL-6 and IL-8, sustained alarmins such as the S100 proteins, and an imbalance of MMPs and TIMPs that mirrors other progressive fibrotic disorders [2,4,8]. An adaptive arm contributes in parallel: a T helper 17-polarized response, promoted by IL-6 at the expense of regulatory T cells, generates interleukin-17A that further drives fibroblast activation and extracellular matrix (ECM) deposition, with regulatory T-cell numbers falling as contracture severity increases [6]. Persistent activation of the IL-6/STAT3 axis has been shown to sustain capsular thickening, collagen deposition and myofibroblast activation in vivo, while mechanotransduction through Piezo1 converts the rising stiffness of the forming capsule into calcium-dependent signaling that promotes the fibroblast-to-myofibroblast transition within a self-reinforcing stiffness–Piezo1–fibrosis loop intersecting the TGF-β and Wnt pathways [5,7]. The relative avascularity of the maturing capsule adds a further profibrotic input through hypoxia and hypoxia-inducible factor 1-alpha (HIF-1α)-driven fibrogenesis [8]. The molecular actions ascribed to PTX, namely PDE inhibition with elevation of intracellular cyclic AMP, suppression of NF-κB signaling, downregulation of SERPINE1 and inhibition of TGF-β1-induced cellular transition, engage several of these nodes simultaneously and provide a coherent, if necessarily indirect, basis for a broad antifibrotic effect expressed at the level of fibroblast proliferation and myofibroblast accumulation [14,15,16,17,18,36,37]. This network perspective positions systemic PTX as a molecular-therapeutic candidate directed at profibrotic signaling, while leaving the precise nodes engaged in vivo to be defined by the mechanistic studies proposed below. Upstream, damage-associated molecular patterns (DAMP) such as heat-shock proteins (HSP) released under mechanical stress, together with subclinical bacterial biofilm, are thought to engage Toll-like receptors and initiate the inflammatory cascade that feeds this network [4].

Translation from the present rat model to the human clinical setting requires acknowledgement of the model’s anatomical and biomechanical constraints. The rat dorsal sub-panniculus carnosus pocket is a low-volume, low-load, glandular-tissue-free environment that does not reproduce the mechanical loading, glandular interface, lymphatic drainage or gravitational forces of the human breast implant pocket, and does not model implant texture or the impact of adjuvant radiotherapy. The findings reported here should therefore be regarded as a controlled signal at the implant–host interface, not as a direct surrogate for human capsular contracture. A structured translational pathway would consist of preclinical confirmation in a sex-balanced, dose-ranging follow-up study with full immunohistochemical and collagen-organization readouts, followed by a randomized, placebo-controlled clinical trial of prophylactic systemic PTX, with or without α-tocopherol, in implant-based breast reconstruction, prioritizing the irradiated high-risk subgroup in whom the prior probability of benefit is highest. For clinical translation the oral route would be the relevant one, as PTX is licensed and in routine use in that form; the intraperitoneal route used here was a preclinical choice to standardize systemic exposure rather than a translational proposal. PTX’s multi-decade safety profile, low cost and broad availability make this a candidate for further translational evaluation.

Several limitations of the present study must be acknowledged transparently and weighed when interpreting the data:

- Histological readout. The histological assessment was restricted to H&E, which was the predefined primary stain of the study protocol and which permitted simultaneous quantification of capsule thickness and stromal cellularity in a single readout. Many modern murine capsule studies combine H&E with Masson’s trichrome and with immunohistochemistry for α-SMA, TGF-β1 and/or Collagen I and III, and a subset additionally use Picrosirius red under polarized light to quantify collagen fiber organization and the collagen I:III ratio [9]. Without α-SMA-specific staining, the present cell counts represent a morphology-based surrogate that cannot reliably distinguish quiescent fibroblasts from activated α-SMA-positive myofibroblasts; the composite term fibroblast/myofibroblast is used throughout to reflect this constraint. Likewise, the absence of Masson’s trichrome and Picrosirius red precludes any conclusion on the qualitative organization of capsular collagen, as distinct from the cellular density and gross thickness quantified here. We consider full immunohistochemical and collagen-organization characterization an essential next step rather than an in-scope extension of the present proof-of-concept evaluation and have structured the proposed follow-up program accordingly.
- Single dose, single timepoint. Only a single PTX dose (25 mg/kg) and a single harvest timepoint at six weeks were used, in line with the predefined proof-of-concept design and the 3R principle of reduction in the absence of prior systematic in vivo data on PTX in a silicone implant model. A controlled dose–response arm with at least two doses bracketing the present regimen, and at least one earlier harvest timepoint capturing the active capsule-formation phase, would have provided stronger pharmacological evidence for a causal effect and would have informed translational dose and timing selection. We do not interpret the present effect estimate as a calibrated dose–response observation, but as a single-point signal at a literature-supported dose. The studies underpinning the dose selection establish tolerability and systemic activity of PTX in the rat at this dose range but are not fibrosis efficacy studies. The present result therefore does not license the inference that 25 mg/kg/day is an optimal or even a fully effective antifibrotic dose, and dose finding is an objective of the proposed follow-up study.
- Single sex. Only male animals were used, in line with the predefined study protocol, to minimize variability associated with the rodent estrous cycle, which is a recognized confounder of fibrotic and wound-healing endpoints in rats. We acknowledge that this design choice is a meaningful limitation in the context of capsular contracture, which affects a predominantly female clinical population, and that a sex-balanced cohort consistent with current Sex and Gender Equity in Research (SAGER) reporting standards is the appropriate next step.
- Thickness measurement. Capsule thickness was quantified at a single standardized site corresponding to the point of maximum macroscopic thickness; the endpoint therefore estimates maximal rather than mean capsule thickness. The rule was fixed in the protocol and applied identically to both groups. Reporting the maximal site alongside a multi-site averaging protocol would be informative in follow-up studies. The cellularity endpoint is unaffected by this consideration, as it was quantified by systematic counting across 10 non-overlapping HPFs rather than by single-site selection.
- Thickness significance and statistical power. The absence of significance for capsule thickness by the inferential test (Student’s t *p* = 0.0588; Mann–Whitney U sensitivity analysis *p* = 0.0452; Cohen’s d = 0.90) reflects the high inter-animal variability of capsule thickness measurements relative to a group size of *n* = 10, with extreme values present in both groups (PTX range 87.7–669.2 µm; control range 241.2–741.5 µm). A post hoc power assessment confirms that the present sample size was adequately powered for the primary cellularity endpoint (achieved power 0.87 at d = 1.47) but clearly underpowered for the secondary thickness endpoint (achieved power 0.48 at d = 0.90); reaching conventional power of 0.80 for the thickness endpoint at the observed effect size would have required approximately *n* = 21 animals per group, while the minimum effect size detectable with the present design at power 0.80 is d ≈ 1.33. We therefore explicitly do not draw a confirmatory conclusion regarding capsule thickness from this study. The 95% CI for the mean difference in thickness crosses zero and the parametric test does not reach the conventional significance threshold; the thickness finding is consequently reported as a directionally consistent but statistically inconclusive secondary signal compatible with, but not establishing, an underlying biological effect, requiring confirmation in an appropriately powered follow-up design.
- No a priori power calculation was performed, and the study may therefore be underpowered to detect differences in secondary endpoints such as capsule thickness. We have addressed this transparently by providing a post hoc power assessment, which confirms that the present design was adequately powered for the primary cellularity endpoint and underpowered for the secondary thickness endpoint.
- Exclusion of hematoma cases, which are known to influence capsule formation, may have influenced both variability and effect estimates and should be considered when interpreting the results. This is of particular relevance because PTX inhibits platelet aggregation. The origin of the hematomas was not investigated, and the sample size does not permit any conclusion regarding bleeding risk. Formal bleeding endpoints are therefore incorporated into the proposed follow-up design.

Despite these limitations, the consistency of the macroscopic findings, the magnitude of the cellularity effect, which was stable in the sensitivity analysis, and the convergence with the established mechanistic profile of PTX together support the value of this proof-of-concept observation as a basis for a structured translational evaluation pathway. We propose a dedicated prospective follow-up study built around four design elements that directly address the limitations of the present work: (i) a controlled dose–response arm with at least two PTX doses bracketing the present 25 mg/kg regimen; (ii) a sex-balanced cohort consistent with current SAGER reporting standards and reflecting the predominantly female clinical target population; (iii) at least two harvest timepoints to capture temporal dynamics of capsule remodeling beyond the single six-week endpoint used here; and (iv) a full mechanistic histopathological readout combining H&E with Masson’s trichrome, α-SMA, TGF-β1 and Collagen I/III immunohistochemistry, and Picrosirius red under polarized light for quantitative collagen-organization analysis, ideally with a textured-implant comparator. Hematoma incidence and formal bleeding endpoints would be recorded prospectively in that design. A relevant downstream translational step would be the controlled clinical evaluation of systemic PTX, with or without α-tocopherol, as a prophylactic adjunct in implant-based breast reconstruction, building on the exploratory single-arm findings reported by Cook et al. [23], ideally within a randomized controlled trial in the irradiated high-risk population in which the prior probability of benefit is highest. Local or topical formulations targeting the pocket interface directly may additionally merit evaluation as an alternative delivery route.

## 4. Materials and Methods

### 4.1. Study Design and Ethical Approval

This was a controlled, single-center experimental animal study conducted at the Experimental Research and Animal Laboratory of Marmara University Faculty of Medicine, Istanbul, Turkey. The protocol was reviewed and approved by the Ethics Committee of Marmara University Faculty of Medicine prior to commencement. All procedures complied with the European Community Council Directive of 24 November 1986 (86/609/EEC) and with Turkish national legislation on the care and use of laboratory animals.

Adherence to the 3Rs principle was addressed where feasible by (i) reduction: a final analyzable group size of *n* = 10 per group after pre-specified exclusions, consistent with comparable historical rat capsule studies; and (ii) refinement: use of a minimized 2 cm × 1 cm subcutaneous implant block to limit surgical burden, single-housing only when necessary to prevent inter-animal injury, daily welfare monitoring, and humane endpoints with deep anesthesia overdose. Replacement of animal experimentation by non-animal alternatives was not feasible for the in vivo capsule biology question addressed, given that the formation of a fibrous peri-implant capsule requires intact wound healing in a vascularized host.

### 4.2. Animals, Housing and Husbandry

Twenty-eight adult male Sprague Dawley rats weighing 250–350 g were sourced and maintained by the Marmara University Experimental Animal Laboratory. Animals were housed individually in standard cages on a 12 h light/dark cycle at constant room temperature, with ad libitum access to standard chow and tap water. Single housing was used to prevent inter-animal trauma to the dorsal surgical site. In line with SAGER reporting recommendations, the sex of the experimental animals is reported transparently: only male Sprague Dawley rats were used in this study, to minimize variability associated with the estrous cycle, which is recognized as a confounder in fibrotic and wound healing endpoints in rodents [2,3].

### 4.3. Group Allocation

Animals were assigned in equal numbers to two groups: Group I (control, intraperitoneal 0.9% saline) and Group II (PTX; Trental^®^, Sanofi-Aventis, Paris, France; 100 mg/5 mL ampoule for injection). Of 28 animals enrolled, 14 were allocated to each group. Four died in the perioperative period, in the context of general anesthesia and the procedure and before the first treatment dose (2 per group). No post-mortem examination was undertaken, and no cause of death was established for individual animals. Four developed peri-implant hematomas detected at explantation, with attendant capsule thickening (2 per group); per a methodological decision consistent with the established literature on hematoma-induced capsule thickening, all 8 animals were excluded from histopathological analysis [39,40]. Twenty animals (10 per group) completed the six-week treatment period and entered analysis.

### 4.4. Implant Preparation

Four 400 cc non-textured (smooth) silicone tissue expanders (Eurosilicone, Apt, France) were sterilized with ethylene oxide in the Marmara University operating theater sterilization unit. Under sterile conditions, the flat dorsal panel of each expander was cut into 2 cm × 1 cm rectangular blocks. Blocks were stored in sterile containers until implantation.

### 4.5. Anesthesia and Surgical Procedure

All surgical procedures were performed under aseptic conditions in the Animal Laboratory operating theater by a single surgeon. After brief diethyl ether inhalational induction, surgical anesthesia was maintained by intraperitoneal injection of ketamine 75 mg/kg (Ketalar^®^, Eczacıbaşı, Istanbul, Turkey) combined with xylazine 10 mg/kg (Rompun^®^, Bayer, Istanbul, Turkey), a standard combination for rat surgical procedures of this duration. Adequacy of anesthesia was confirmed by absence of tail-pinch and limb-withdrawal reflexes.

The dorsal surgical field was clipped, disinfected with povidone-iodine and rinsed with sterile saline. A 3 cm vertical incision was made over the thoracodorsal region. By blunt dissection, a 3 cm × 2 cm pocket was developed beneath the panniculus carnosus, and one sterile silicone block was inserted per animal. Incisions were closed in two layers, with subcutaneous 3/0 Vicryl Rapide^®^ (Ethicon, Norderstedt, Germany) and cutaneous 2/0 Mersilk^®^ (Ethicon, Norderstedt, Germany). Animals were returned to single-cage housing. Postoperative wound healing, body weight, food and water intake, dehiscence, infection and mortality were monitored daily by an investigator blinded to group allocation.

### 4.6. Treatment Protocol

Beginning on the first postoperative day, animals received once-daily intraperitoneal injections at the same time each day. Group I received 1 mL sterile 0.9% saline. Group II received PTX (Trental^®^, 100 mg/5 mL) at 25 mg/kg in a 1 mL volume. The intraperitoneal route was selected for the preclinical setting because it guarantees delivery of an exact, body-weight-adjusted dose to every animal on every treatment day, avoids the variable and incomplete absorption associated with oral administration, and thereby standardizes systemic exposure across a six-week treatment period. The alternatives of daily oral gavage and drinking-water administration would have introduced, respectively, repeated restraint stress with a risk of aspiration and uncontrolled per-animal intake. The 25 mg/kg dose lies within the range of daily doses administered systemically to rats in published studies, in which it was tolerated without dose-limiting systemic toxicity [41,42,43,44,45,46]. Treatment continued for six weeks, the interval after which a mature peri-implant capsule reliably forms in the rat [24]. A single fixed dose was used to test the primary hypothesis under standardized conditions. Dose–response characterization, including the higher doses and alternative systemic routes described in the rat, is reserved for subsequent work [47].

### 4.7. Euthanasia and Tissue Harvest

On postoperative day 42, all animals were euthanized by intraperitoneal pentobarbital 150 mg/kg overdose. The implant and surrounding capsule were excised en bloc together with the overlying skin and immersed in 10% buffered formaldehyde for 7 days of fixation. After fixation, the silicone block and capsule were dissected at the Marmara University Pathology Laboratory under macroscopic examination. Two paraffin blocks were prepared per capsule: the first contained a transverse slice taken through the point of maximum macroscopic thickness for thickness measurement; the second contained the remainder of the capsule for cell counting.

### 4.8. Histopathological Analysis

Sections of 5 µm were cut from each paraffin block with a microtome and stained with H&E. H&E was selected as the primary stain because it permits simultaneous quantification of capsule thickness and total stromal cellularity in a single readout, which were the two pre-specified endpoints of this study.

Capsule thickness was measured on the first slide of each animal using an Olympus BX51 light microscope (Olympus Corporation, Tokyo, Japan) coupled to the analySIS five digital imaging solution (Olympus Soft Imaging Solutions, Münster, Germany). Measurements were taken at the point where the capsule lay fully flat on the slide, corresponding to the maximum macroscopic capsule thickness for each animal, and recorded in micrometers. The requirement that the capsule lie fully flat is a technical one: obliquely or tangentially sectioned capsule cannot be measured without overestimation of thickness. The endpoint quantified by this protocol is therefore the maximal capsule thickness at a standardized site, not the mean thickness averaged over the capsule surface. The sampling rule was fixed in the protocol and applied identically to both groups.

On the second slide of each animal, total fibroblast/myofibroblast counts were performed in 10 non-overlapping HPFs at 40× objective magnification, and the per-animal mean was used in subsequent analyses. All cells with fusiform morphology consistent with fibroblast or myofibroblast lineage were included; the composite term fibroblast/myofibroblast count is used throughout to denote this morphology-based readout, with α-SMA-specific identification reserved for follow-up immunohistochemical work. Sections were evaluated and systematically graded in a random order by a blinded, independent pathologist.

### 4.9. Statistical Analysis

Two pre-specified endpoints were analyzed: intracapsular fibroblast/myofibroblast density and peri-implant capsule thickness. No formal endpoint hierarchy was specified in the original protocol. For the present report, intracapsular fibroblast/myofibroblast density is designated the primary endpoint and capsule thickness the secondary endpoint on mechanistic grounds: cellularity is the proximate cellular readout of the hypothesized antifibrotic action of PTX, whereas thickness is a downstream, multifactorial tissue-level consequence of it.

The parametric two-sample Student’s *t*-test is the inferential test for both endpoints; it was chosen because the interpretation rests on the difference in group means with its confidence interval, because the equal group sizes support the robustness of the test, and because its assumptions were satisfied (Shapiro–Wilk *p* > 0.05 in all four datasets; Levene’s test *p* = 0.687 for thickness and *p* = 0.210 for cell count). The non-parametric Mann–Whitney U test is reported alongside as a sensitivity analysis, in a subordinate role: it indicates whether the inference is stable under weaker distributional assumptions, and no conclusion is drawn from it.

Effect sizes are reported as Cohen’s d using the pooled standard deviation, and mean differences are accompanied by 95% confidence intervals derived from Welch’s approximation. Two-sided *p* < 0.05 was considered statistically significant. Data are presented as mean ± standard deviation, with minimum, maximum and median values reported in tabular form. The group size of *n* = 10 per group is consistent with comparable historical murine peri-implant capsule studies, predominantly conducted in rat models [9,24]. No formal a priori power calculation was performed for the present study; this is acknowledged as a limitation in the Discussion. A post hoc power assessment was performed using the observed effect sizes, the per-group sample size of n = 10 and two-sided α = 0.05. Achieved power was 0.87 for cellularity (d = 1.47) and 0.48 for capsule thickness (d = 0.90). The minimum effect size detectable with the present design at conventional power of 0.80 is d ≈ 1.33; detection of an effect size of d = 0.90 at conventional power of 0.80 would have required approximately *n* = 21 animals per group. All statistical analyses and figures were performed using Python 3.12 (Python Software Foundation, Wilmington, DE, USA) with standard statistical and data visualization libraries.

## 5. Conclusions

Six weeks of daily systemic PTX at 25 mg/kg significantly reduced intracapsular fibroblast/myofibroblast density around smooth silicone blocks in a controlled rat model (Cohen’s d = 1.47, *p* = 0.0040). A directionally consistent but statistically inconclusive reduction in capsule thickness of comparable magnitude was observed as a secondary signal, requiring confirmation in an appropriately powered follow-up design. Given the pivotal role of fibroblastic activity in driving myofibroblast-mediated capsule contraction, and given PTX’s well-established multi-decade safety profile, low cost and broad availability, the present findings support a structured translational evaluation pathway: dose–response and mechanistic preclinical follow-up with collagen and immunohistochemical readouts, and cautious translational evaluation of PTX as a low-cost antifibrotic adjunct in implant-based reconstructive surgery, within the limitations of an H&E-only, single-dose, single-sex design.

## Figures and Tables

**Table 1:** Summary statistics and group comparisons for fibroblast/myofibroblast density and capsule thickness.

Parameter	Cell Density (Cells/HPF)	Capsule Thickness (µm)
PTX, mean ± SD (*n* = 10)	36.28 ± 9.80	340.10 ± 199.88
Control, mean ± SD (*n* = 10)	56.46 ± 16.69	507.78 ± 170.72
PTX, median	36.10	268.97
Control, median	51.40	513.40
PTX, range	20.20–49.20	87.70–669.16
Control, range	29.80–82.80	241.19–741.47
Mean difference (95% CI)	20.18 (7.10–33.26)	167.69 (−7.26 to 342.63)
Reduction (%)	35.7%	33.0%
Cohen’s d	1.47	0.90
Student’s *t*-test, *p*	0.0040	0.0588
Mann–Whitney U test, *p*	0.0058	0.0452

Values are mean ± standard deviation; range and median are also given. Mean differences are reported with 95% confidence intervals derived from Welch’s approximation. Group comparisons by unpaired Student’s *t*-test, with the Mann–Whitney U test reported as a sensitivity analysis. Effect sizes calculated as Cohen’s d using the pooled standard deviation.

## Data Availability

All data relevant to the conclusions of this study are presented within the manuscript (Table 1). Additional supporting data is available from the corresponding author upon request.

## Abbreviations

The following abbreviations are used in this manuscript:

α-SMA	Alpha smooth muscle actin
ADM	Acellular dermal matrix
ADSC	Adipose-derived stem cell
CI	Confidence interval
DAMP	Damage-associated molecular pattern
ECM	Extracellular matrix
H&E	Hematoxylin and eosin
HIF-1α	Hypoxia-inducible factor 1-alpha
HPF	High-power field
HSP	Heat-shock protein
IL-1β	Interleukin-1 beta
IL-6	Interleukin-6
IL-8	Interleukin-8
LTRA	Leukotriene receptor antagonist
MMP	Matrix metalloproteinase
NF-κB	Nuclear factor kappa B
PDE	Phosphodiesterase
PDGF	Platelet-derived growth factor
PTX	Pentoxifylline
SAGER	Sex and Gender Equity in Research
SERPINE1	Serpin family E member 1 (Plasminogen activator inhibitor-1)
TGF-β1	Transforming growth factor beta 1
TIMP	Tissue inhibitor of metalloproteinases
TNF-α	Tumor necrosis factor-alpha
